# Impact of Adjunctive Air-Polishing on Periodontal Status in Patients with Low Vitamin D_3_ Levels: A Retrospective Study

**DOI:** 10.3390/jcm14248775

**Published:** 2025-12-11

**Authors:** Alexandra Cornelia Teodorescu, Elena-Raluca Baciu, Alice Murariu, Ionuț Luchian, Irina Georgeta Șufaru, Liliana Păsărin, Bogdan Constantin Vasiliu, Gabriel Rotundu, Sorina Mihaela Solomon

**Affiliations:** Faculty of Dental Medicine, Grigore T. Popa University of Medicine and Pharmacy, 700115 Iasi, Romania; cornelia.teodorescu@umfiasi.ro (A.C.T.); alice.murariu@umfiasi.ro (A.M.); ionut.luchian@umfiasi.ro (I.L.); ursarescu.irina@umfiasi.ro (I.G.Ș.); liliana.pasarin@umfiasi.ro (L.P.); bogdan.vasiliu@umfiasi.ro (B.C.V.); gabriel.rotundu@umfiasi.ro (G.R.); sorina.solomon@umfiasi.ro (S.M.S.)

**Keywords:** erythritol, glycine, periodontitis, vitamin D_3_

## Abstract

**Background/Objectives**: Air-polishing has become, in recent years, a very popular additional tool to subgingival debridement for treating periodontal disease. Vitamin D_3_ plays a crucial role in bone metabolism and calcium-phosphate homeostasis. The aim of our retrospective study was to determine the additional effect of subgingival air-polishing with two types of powders (glycine and erythritol) on patients with different stages of periodontitis and low serum levels of Vitamin D_3_. **Methods**: We collected and analysed the data of 62 patients (demographics, vitamin D_3_ levels, plaque index, periodontal probing depth, bleeding on probing, clinical attachment loss, periodontitis stage and type of air-polishing powder) used during periodontal therapy. **Results**: We did not observe a significant correlation between periodontal status and vitamin D_3_ levels/status (mean Vitamin D_3_ levels: Stage I—20.19 ± 4.413 ng/mL; Stage II—19.482 ± 3.814 ng/mL; Stage III—17.681 ± 5.869 ng/mL; Stage IV—17.578 ± 5.94 ng/mL; and *p* = 0.539), nor did we find any significant differences in clinical outcomes when using glycine or erythritol in addition to scaling and root planning (SRP) at 3 months after treatment (all *p* > 0.05). **Conclusions**: The discrete association between lower levels of vitamin D_3_ and more advanced stages of periodontitis could suggest a possible influence of vitamin D_3_ insufficiency on periodontal disease progression. Although safe, easy to use and comfortable for patients, glycine and erythritol showed no differences in periodontal clinical parameters when compared as an addition to SRP.

## 1. Introduction

Periodontitis, the sixth most common oral illness worldwide, is a chronic, multifactorial inflammatory disease characterised by the progressive destruction of the periodontal ligament and alveolar bone [1,2,3]. Its aetiology is linked to a combination of local and systemic factors, including dysbiotic and anaerobic microbial biofilm—such as *Porphyromonas gingivalis*, *Tannerella forsythia*, and *Treponema denticola*—as well as morphological alterations of tooth surfaces, dental arch irregularities, temporomandibular joint dysfunction, occlusal or masticatory imbalances, and systemic health conditions [4,5]. Clinical manifestations of periodontitis include gingival inflammation, the occurrence of periodontal pockets, bleeding on probing, clinical attachment loss, loss of bone as seen by radiographs, mobility, and, in later stages, pathologic migration leading to tooth loss [1,2,5,6].

Cholecalciferol (Vitamin D_3_) is a fat-soluble secosteroid, primarily synthesised in the skin through ultraviolet B exposure and supplemented by dietary intake, that plays an essential role in regulating calcium–phosphate homeostasis and mineral bone metabolism [7,8,9,10,11,12]. It also plays an essential role in muscle strength, autoimmune disease and diabetes [10]. Vitamin D3 deficiency has been linked to increased risk of fractures, insulin resistance, cardiovascular disease, inflammatory bowel disease, neurodegenerative disease such as Parkinson’s and Alzheimer’s dementia and even some types of cancer [7,8,10,11].

Recent studies and systematic reviews have reported associations between low serum 25-hydroxy vitamin D_3_ [25(OH)D_3_] concentrations—typically defined as <20 ng/mL—and chronic periodontitis, with an increased bleeding on probing, whereas higher serum levels appear to correlate with reduced inflammation and improved periodontal stability [13,14,15,16,17,18,19,20,21]. According to experimental data, vitamin D_3_ may exert protective effects by inhibiting *Porphyromonas gingivalis* growth by activating autophagy and reducing inflammation in periodontal tissues [22]. However, there is still conflicting data about the connection between periodontal disease and vitamin D_3_ levels.

Given that biofilm removal is an essential aspect of periodontal treatment guidelines, air-polishing devices have become a viable supplement to conventional supragingival and subgingival instrumentation. These devices deliver a controlled mixture of water, compressed air, and abrasive particles to effectively disrupt and remove subgingival biofilm. They are considered a safe, comfortable, and time-efficient adjunct to periodontal therapy, complementing hand instrumentation during initial treatment and serving as a stand-alone approach for managing residual pockets or performing supportive periodontal therapy [23].

Despite their effectiveness on enamel, the abrasiveness of sodium bicarbonate powders renders them unsuitable for use on exposed root surfaces. The risk of hard tissue loss is mitigated by low-abrasive polishing powders (LAPPs), particularly those derived from erythritol (a non-cariogenic, well-tolerated, and non-metabolised sugar alcohol) or glycine (an amino acid), which possess smaller particle sizes, reduced hardness, and enhanced water solubility [2,24,25,26]. Several systematic reviews have compared the efficacy of erythritol and glycine as subgingival air-polishing powders during initial periodontal therapy, in the management of residual pockets, and within supportive periodontal care. These analyses reported no significant differences between the two powders in terms of clinical improvements (such as reductions in PPD, CAL, and BOP) or in their effectiveness for biofilm control [23,27,28].

Numerous studies have explored the association between low serum vitamin D_3_ levels and periodontitis, typically comparing periodontally healthy individuals with patients diagnosed with chronic or aggressive forms of the disease [12,13,14,17]. Most of these investigations have reported significantly lower vitamin D_3_ levels in patients with periodontitis than in healthy controls, suggesting that vitamin D_3_ deficiency may play a role in periodontal disease susceptibility. In light of these findings, the aim of this study was to evaluate the effect of subgingival air-polishing with glycine or erythritol powder as reflected across the four stages of periodontitis (as defined by the 2018 classification [29]) of patients with low vitamin D3 levels.

To address this objective, these null hypotheses were formulated:

- There are no significant differences in the distribution of periodontitis stages, and no associations exist between baseline periodontal parameters and serum vitamin D3 levels;
- There are no significant differences in clinical periodontal parameters (plaque index, probing pocket depth, bleeding on probing, and clinical attachment loss) between patients treated with subgingival air-polishing using glycine powder and those treated with erythritol powder.

## 2. Materials and Methods

### 2.1. Research Design and Criteria for Patient Selection

The datasets from adult patients with periodontal pathologies were retrospectively collected between March 2024 and May 2025 from the archives of a private clinic in Iasi, Romania, in collaboration with the Department of Periodontics, School of Dentistry, “Grigore T. Popa” University of Medicine and Pharmacy in Iasi, Romania. The study was approved by the Ethics Committees of “Grigore T. Popa” University of Medicine and Pharmacy in Iasi with registration number 635/27.08.2025. All procedures were carried out in accordance with the ethical standards outlined in the 1964 Declaration of Helsinki and its subsequent amendments (2013).

### 2.2. The Study Group

The clinical records of 106 patients diagnosed with stage I, II, III, or IV periodontitis, according to the 2018 classification, were reviewed. Of these, 62 cases (56.85%) met the inclusion criteria, presenting with low serum vitamin D3 levels—classified as deficiency (<20 ng/mL) or insufficiency (20–29.9 ng/mL) [16].

Inclusion criteria consisted of

- Low serum levels of vitamin D3;
- Non-smokers;
- Diagnosis of periodontitis (including stage of disease);
- Ages between 18 years and 50 years;
- A minimum of twenty teeth present for each patient.

The staging of periodontitis used in the study group was as follows [29]:

- Stage I: 1–2 mm interdental CAL (clinical attachment loss), with maximum probing depth of 4 mm, mostly horizontal bone loss and no teeth lost due to periodontitis;
- Stage II: 3–4 mm interdental CAL, with maximum probing depth of 5 mm, mostly horizontal bone loss and no teeth missing due to periodontitis;
- Stage III: ≥5 mm interdental CAL at the site of greatest loss, probing depth of at least 6 mm, a maximum of 4 periodontitis-related teeth lost; can associate vertical bone loss ≥3 mm and furcation involvement class II or III;
- Stage IV: in addition to stage 3, this stage of periodontitis consists of ≥5 teeth lost due to periodontitis and a need for complex rehabilitation due to masticatory dysfunction, secondary occlusal trauma or bite collapse and drifting and flaring of the teeth.

The remaining 44 cases were excluded due to the following criteria:

- Incomplete dataset;
- Habit of smoking 3 years prior to examination, controlled substance abuse;
- Ages older than 50 years;
- Menopausal women;
- Having fewer than 20 teeth or wearing fixed orthodontic appliances;
- Taking antibiotics in the past 3 months;
- Taking anti-inflammatory drugs in the past 4 weeks;
- Patients with a history of vitamin D_3_ supplementation;
- Receiving periodontal therapy within the past 6 months;
- Failing to come to the 3-month follow-up.

Subsequently, patients were categorised into four main groups based on their periodontal status, and the data extracted from their clinical records were organised as follows:

- Demographic variables, such as age and sex;
- Baseline serum 25-hydroxyvitamin D_3_ levels;
- Periodontal charting at baseline (T0) and three months post-treatment (T1), which included the plaque index (PI), periodontal probing depth (PPD), bleeding on probing (BOP) and clinical attachment loss (CAL);
- Periodontitis stages;
- Details of the administered periodontal therapy;
- Photographic documentation obtained before, during, and after treatment.

### 2.3. Clinical Evaluation and Non-Surgical Periodontal Treatment Protocol

Being a retrospective study, we gathered the serum concentrations of 25(OH)D_3_ from the patients’ clinical records. They were determined based on fasting blood samples collected within 7 days from each baseline periodontal evaluation.

Periodontal charting, at baseline and three months post-treatment, included plaque index (PI), periodontal probing depth (PPD), bleeding on probing (BOP) and clinical attachment loss (CAL), and was performed by one experienced, certified periodontist (A.C.T.).

For the PI, a disclosing agent (Rondells Blue, Directa, Upplands Vasby, Sweden) was used on all the lateral aspects of the teeth. The presence of coloured plaque was counted as a “1” and the absence as “0”. The sum of all coloured lateral aspects of the teeth was divided by the total number of examined surfaces and multiplied by 100, thus giving the PI percentage (%).

In a similar manner, the BOP was determined with the help of a periodontal probe (North Carolina) by gently probing every lateral aspect of each tooth. The sum of all bleeding surfaces was divided by the total number of examined surfaces and multiplied by 100, thus obtaining the BOP (%).

Using the same periodontal probe, PPD was calculated for each tooth as the distance between the junctional epithelium and the gingival margin and CAL—the distance between the junctional epithelium and the CEJ (cementum–enamel junction).

**Figure 1:**
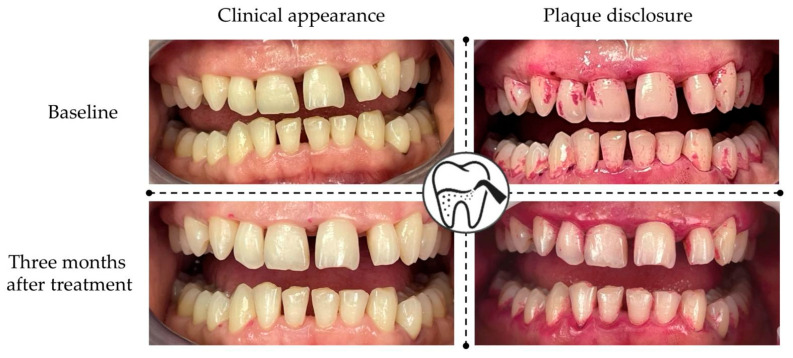
Clinical aspect of a stage II periodontitis patient treated with SRP and erythritol subgingival air-polishing (case belonging to Dr Alexandra Cornelia Teodorescu).

As a periodontal treatment protocol, all patients received a full-mouth scaling and root planning without the use of antiseptics prior to our data collection. They were per-formed by the same periodontist (A.C.T.—Figure 1) using hand instruments (Gracey mini curettes—LM Dental, Parainen, Finland) and ultrasonic scalers (Varios 350—NSK, Tochigi, Japan, with Varios G1 and P10 Perio tips—NSK, Tochigi, Japan), completed with subgingival air-polishing with glycine (AirFlow Perio—EMS Dental, Nyon, Switzerland) or erythritol powder (AirFlow Plus—AirFlow Perio—EMS Dental, Nyon, Switzerland), powered by an Air-N-Go Easy handpiece with a Perio Easy tip (Acteon, Merignac, France). The patients’ clinical records had no mention of any complications occurring during the treatment phase (subcutaneous emphysema or post-operative pain).

### 2.4. Statistical Analysis

All statistical analyses were performed using IBM SPSS Statistics software (version 26.0; IBM Corp., Armonk, NY, USA). The sample size calculation was performed using the online tool available at Calculator.net. The analysis was based on a significance level of 0.05 and a statistical power of 80%, which indicated that a minimum of 59 patients were required for the study. Normality of data distribution was determined using the Shapiro–Wilk test. As the data did not follow a normal distribution, non-parametric tests were applied. Intra-group comparisons between baseline and 3-month values were evaluated using the Wilcoxon signed-rank test, while inter-group comparisons were analysed using the Mann–Whitney U and Kruskal–Wallis tests. Associations between categorical variables (vitamin D_3_ status and periodontitis stage) were assessed using the chi-square test. A *p*-value less than 0.05 was considered statistically significant.

## 3. Results

### 3.1. Descriptive

**Figure 2:**
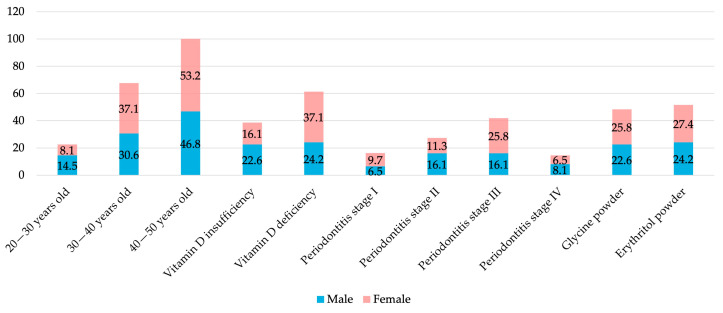
Distribution of study participant characteristics according to gender.

The study included a total of 62 patients (29 males and 33 females), with ages ranging from 22 to 50 and an average age of 42.6 years. The mean serum 25(OH)D_3_ concentration was 18.56 ng/mL, with 38 patients (61.3%), including 23 females (69.7%), diagnosed with vitamin D_3_ deficiency (<20 ng/mL). Regarding periodontal status, most patients were diagnosed with stage III periodontitis, of whom 25.8% were females. As for the periodontal therapy, 30 patients received additional subgingival air-polishing with glycine powder, while 32 were treated with erythritol powder (Table 1, Figure 2).

Analysis of clinical parameters by periodontitis stage revealed statistically significant improvements for all periodontal parameters from baseline to 3 months (*p* < 0.001), with very large effect sizes (r = 0.85–0.87), indicating great treatment-related changes across the study participants (Table 2).

### 3.2. Testing the First Hypothesis

**Figure 3:**
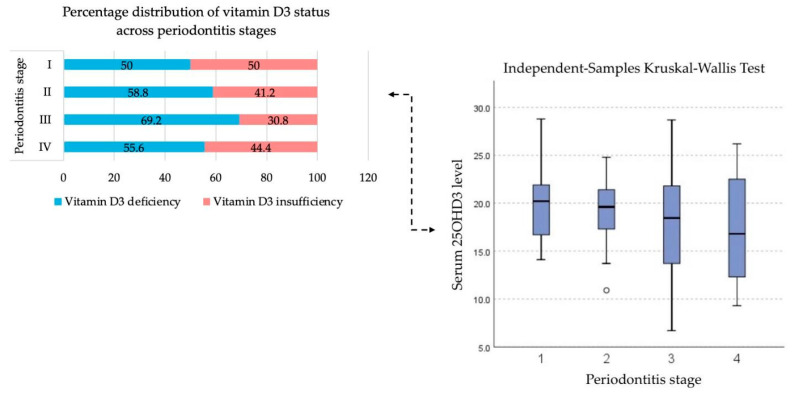
Distribution of baseline serum vitamin D_3_ concentrations across different stages of periodontitis.

No significant association was found between serum vitamin D_3_ levels and periodontitis stage (*p* = 0.539—Table 3), although there is a tendency for the vitamin D_3_ levels to be lower as the periodontitis progresses to more severe stages with a median of 20.5 ng/mL—stage I, 19.6 ng/mL—stage II, 18.45 ng/mL—stage III and 16.80 ng/mL—stage IV. It was also observed that patients with stage III periodontitis exhibited a higher prevalence of vitamin D_3_ deficiency compared with those in the other stages (Figure 3).

Similarly, baseline periodontal parameters (PI, PPD, BOP, CAL) showed no statistically significant differences (all *p* > 0.05) when comparing patients with vitamin D_3_ deficiency to those with insufficiency (Table 4).

### 3.3. Testing the Second Hypothesis

Although the group treated with subgingival air-polishing using erythritol powder showed minor improvements in PI, BOP, and CAL when compared to the glycine group, no statistically significant differences were observed between the two groups at 3 months (all *p* > 0.05) (Table 5).

## 4. Discussion

The present study aimed to evaluate the clinical benefits of erythritol or glycine as adjunctive subgingival air-polishing agents in non-surgical periodontal treatment. This evaluation focused on patients with varying stages of periodontitis and low serum vitamin D_3_ levels.

### 4.1. Vitamin D_3_ Levels and Periodontal Status

In evaluating the first hypothesis, no statistically significant association was identified between serum vitamin D_3_ status and periodontitis staging, nor between baseline periodontal parameters and vitamin D_3_ categories (deficiency or insufficiency). Vitamin D_3_ deficiency was defined as serum levels < 20 ng/mL, whereas insufficiency corresponded to levels between 20 and 29.9 ng/mL. Although patients in more advanced stages of periodontitis tended to exhibit lower vitamin D_3_ levels, this trend did not reach statistical significance.

This link between levels of vitamin D_3_ and the severity of periodontitis has been a topic that sparked interest in the last few years in the medical field. But the results of the published studies are conflicting [13,17,30]. It is also important to note that many previously published studies classified patients according to the former periodontitis classification system, which limits the comparability of their results with those of the present study.

Earlier studies have attempted to clarify the relationship between periodontitis and vitamin D3 deficiency. A Norwegian investigation conducted in 2019 assessed serum 25(OH)D_3_ concentrations in ethnic Norwegian and Tamil refugee patients, both with and without periodontitis. The authors reported that lower vitamin D_3_ levels were associated with the presence of periodontitis only in the Norwegian cohort, while no significant differences were observed among Tamil participants [17].

Similarly, in 2018, Anbarcıoğlu et al. [13] compared individuals with aggressive or chronic periodontitis to periodontally healthy controls. Their findings indicated that patients with aggressive periodontitis exhibited lower serum vitamin D_3_ levels than those with chronic periodontitis or healthy individuals, although no statistically significant differences were detected between the chronic periodontitis group and healthy controls [13].

Additional studies have examined vitamin D_3_ status in patients with moderate to severe periodontitis [30] or severe periodontitis [31] relative to healthy subjects. Both studies reported reduced vitamin D_3_ levels among periodontitis patients; however, Laky et al. [31] found no significant correlations between vitamin D_3_ concentration and key periodontal parameters, including PPD, CAL, and BOP.

Contrary to our findings, studies published in 2022 and 2024 found that the stage of periodontitis (I–IV) was significantly negatively correlated with the serum level of vitamin D_3_. The vitamin D_3_ level decreased with the progression of the disease to a higher stage [32,33].

One extensive Northern study found that vitamin D_3_ status was associated with periodontitis stages II–III/IV. People with periodontitis stages II-III/IV had 53% higher odds of having deficient vitamin D_3_ levels than those in the non-periodontitis/stage I group [34].

However, there is one other study that did not find any association between vitamin D_3_ levels and periodontal diseases, gingival inflammation to be exact, consistent with our present findings [35]. Several factors can induce vitamin D_3_ deficiency, including systemic health problems affecting the metabolism of vitamin D_3_, limited sun exposure and dietary deficiency [36].

It is important to acknowledge that serum vitamin D_3_ levels are subject to seasonal variation, primarily due to fluctuations in sunlight exposure throughout the year [34]. As the patient data in the present study were collected over a one-year period, this seasonal influence may have introduced variability in the recorded vitamin D_3_ concentrations.

### 4.2. Subgingival Air-Polishing

When looking at the additional effect of subgingival air-polishing agents such as glycine and erythritol, we found that, although all the periodontal parameters improved at 3 months after the periodontal therapy, there were no statistically significant differences between the glycine or erythritol groups.

With a particle size of 25–65 μm, glycine is a frequently used air-polishing agent that can reduce the patients’ pain and discomfort during periodontal therapy. It is highly soluble, bio-compatible and has the advantage that it can disrupt the dental biofilm without damaging the hard teeth surfaces or soft tissues surrounding them [37]. It can also have an effect on reducing the subgingival bacterial overgrowth of *P. gingivalis*, *A. actinomycetemcomitans* and *F. nucleatum* [38].

Erythritol has a particle size of 14 μm, which is smaller and less abrasive when compared to that of glycine powder. Its advantages include good stability and water-soluble properties, and it is used outside the medical field as an artificial sweetener and food additive [37].

At the present time, there are not many studies investigating the effects of glycine or erythritol on the periodontal therapy outcomes when associated with scaling and root planning (SRP). Two different meta-analyses found that there were no significant statistical differences in the improvement of periodontal parameters such as PPD, CAL and BOP, or biofilm control by three subgingival air-polishing powders: erythritol, glycine or trehalose [23,28]. Their findings were consistent with what we have also found regarding glycine versus erythritol as an additional tool to SRP.

Erythritol-focused studies have shown that the agent provides clinical advantages during initial non-surgical periodontal therapy, particularly in cases presenting with deep periodontal pockets [25,28,39]. Conversely, glycine has demonstrated short-term benefits in reducing subclinical inflammation, as indicated by the volume of gingival crevicular fluid [40]. However, when used in conjunction with scaling and root planning (SRP), glycine produced similar clinical, inflammatory, and microbial outcomes to SRP alone [38].

The available speciality literature lacks clinical in vivo studies directly comparing the efficacy of adjunctive glycine and erythritol powders when used in combination with SRP. In 2021, Seidel et al. published an in vitro study that found that power scalers achieved significantly higher relative cleaning efficacy than air-polishing devices for the furcation area, with glycine application with a subgingival nozzle having similar results with ultrasonic scalers. The advantage of the air-polishing systems was the shorter treatment time [41].

In the context of subgingival air-polishing procedures, it is essential to acknowledge the potential risk of subcutaneous emphysema [42,43]. Each dental professional using an air-polishing device should be capable of making a rapid subcutaneous emphysema diagnosis and should know the right clinical approach based on the severity and the patient’s medical history.

### 4.3. Study Limitations

The present study has certain limitations, including the retrospective nature of the analysis, a limited sample size (which may increase the risk of type II error), and the absence of control groups for the hypotheses investigated (no group with sufficient vitamin D3 levels for the first hypothesis; no SRP-only group for the second one). Future studies should therefore include larger, multicentre cohorts, employ standard measurement protocols, extend follow-up periods, and account for seasonal variations in vitamin D3 synthesis, either by controlling for these factors or by standardising the timing of sample collection to minimise potential confounding effects.

## 5. Conclusions

Within the limitations of this study, no causal relationship between vitamin D_3_ deficiency and periodontitis could be demonstrated. Nevertheless, the observed association between lower levels and more advanced stages of the disease suggests a possible contributory role of vitamin D_3_ insufficiency in periodontal deterioration. Recognising this potential association may support the development of more personalised preventive strategies and tailored periodontal therapy.

Oral Vitamin D_3_ supplementation should be used for all individuals presenting with low vitamin D_3_ deficiencies, and all periodontitis patients should have their serum levels tested. This approach could provide a more holistic treatment for all patients.

In relation to the adjunctive use of erythritol and glycine in non-surgical periodontal treatment, both subgingival powders have demonstrated safety and patient acceptability. The biofilm disruption by mechanical means is the “golden rule” for periodontitis treatment; thus, combining different treatment approaches could mean better clinical outcomes. However, more studies are required in order to determine whether their application provides a significant therapeutic advantage over conventional approaches and to evaluate whether their cost–benefit ratio justifies routine clinical use.

Overall, the findings from this study reinforce the importance of conventional non-surgical therapy as the foundation of periodontal treatment and indicate that, while vitamin D_3_ deficiency is common among periodontitis patients, its clinical influence requires further exploration.

## Figures and Tables

**Table 1:** Characteristics of study participants (N = 62).

**Variable**	**Patient Characteristics**	**Count (N)**	**Percentage (%)**
	***Gender***	(Mean ± SD)	
	Male	29	46.8
	Female	33	53.2
	***Age***		
	20–30 years old	6	9.7
	31–40 years old	14	22.6
	41–50 years old	42	67.7
	Mean ± SD (Minimum–Maximum)	42.60 ± 7.09 (22–50)	
	***Low serum 25OHD3 level*** **(ng/mL)**		
	Vitamin D_3_ insufficiency	24	38.7
	Vitamin D_3_ deficiency	38	61.3
	Mean ± SD (Minimum–Maximum)	18.56 ± 5.15 (6.7–28.8)	
	***Periodontitis***		
	Stage I	10	16.1
	Stage II	17	27.4
	Stage III	26	41.9
	Stage IV	9	14.5
	***Subgingival air-polishing***		
	Glycine powder	30	48.4
	Erythritol powder	32	51.6

SD—Standard deviation.

**Table 2:** Clinical parameters of study participants (N = 62).

Clinical Parameters		Periodontitis Stage (Mean ± SD)				Related-Samples Wilcoxon Signed-Rank Test
		I (N = 10)	II (N = 17)	III (N = 26)	IV (N = 9)	
PI (%)	Baseline	95.80 ± 8.12	88.82 ± 26.02	94.08 ± 13.15	85.67 ± 14.41	Z = −6.853; r = 0.87 *p* < 0.001 *
	3 months	23.10 ± 32.73	26.76 ± 22.88	27.15 ± 21.46	54.22 ± 14.77	
PPD (mm)	Baseline	3.60 ± 0.58	4.10 ± 0.86	4.80 ± 0.97	3.98 ± 0.84	Z = −6.792; r = 0.86 *p* < 0.001 *
	3 months	2.09 ± 0.32	2.42 ± 0.43	2.71 ± 0.52	3.01 ± 0.52	
BOP (%)	Baseline	95 ± 8.99	86.24 ± 21.30	89.73 ± 19.03	74.22 ± 24.05	Z = −6.855; r = 0.87 *p* < 0.001 *
	3 months	22.10 ± 24.37	17.47 ± 15.95	26.15 ± 13.89	25.78 ± 14.61	
CAL (mm)	Baseline	1.50 ± 0.63	2.85 ± 0.84	4.33 ± 1.76	2.98 ± 0.64	Z = −6.682; r = 0.85 *p* < 0.001 *
	3 months	0.78 ± 0.43	1.77 ± 0.68	2.78 ± 1.20	2.15 ± 0.72	

* *p* < 0.05; SD—Standard deviation; PI—plaque index; PPD—periodontal probing depth; BOP—bleeding on probing; CAL—clinical attachment loss.

**Table 3:** Association between periodontitis stage and serum vitamin D3 levels.

Clinical Parameter	Periodontitis Stage (Mean ± Standard Deviation)				Kruskal–Wallis Test
	I (N = 10)	II (N = 17)	III (N = 26)	IV (N = 9)	
Serum 25OHD3 level ng/mL	20.19 ± 4.413	19.482 ± 3.814	17.681 ± 5.869	17.578 ± 5.94	*p* = 0.539

**Table 4:** Association between baseline periodontal clinical parameters according to vitamin D3 status.

Baseline Clinical Parameters	Vitamin D_3_ Insufficiency N = 24		Vitamin D_3_ Deficiency N = 38		Mann–Whitney Test
	Mean ± SD	Min–Max	Mean ± SD	Min–Max	
PI (%)	90 ± 22.39	5–100	92.76 ± 13.30	54–100	U = 451.500, Z = −0.080, r = 0.010 *p* = 0.936
PPD (mm)	4.29 ± 1.04	3–6.25	4.30 ± 0.94	2.33–6.42	U = 483.000, Z = 0.390, r = 0.050 *p* = 0.696
BOP (%)	86.50 ± 20.66	33–100	87.92 ± 19.82	33–100	U = 477.500, Z = 0.354, r = 0.045 *p* = 0.723
CAL (mm)	3.01 ± 1.49	0.67–5.92	3.44 ± 1.69	0.42–6.92	U = 477.500, Z = 0.745, r = 0.095 *p* = 0.723

SD—Standard deviation; Min—Minimum; Max—Maximum; PI—plaque index; PPD—periodontal probing depth; BOP—bleeding on probing; CAL—clinical attachment loss.

**Table 5:** Association between periodontal clinical parameters according to the type of subgingival air-polishing.

Clinical Parameters	Subgingival Air-Polishing					
	Glycine Powder (N = 30)			Erythritol Powder (N = 32)		
	Baseline (T0) Mean ± SD	At 3 Months (T1) Mean ± SD	ΔT = T0 − T1 Mean ± SD	Baseline (T0) Mean ± SD	At 3 Months (T1) Mean ± SD	ΔT = T0 − T1 Mean ± SD
PI (%)	94.28 ± 11.32	29.50 ± 24.16	64.78 ± 28.55	88.93 ± 21.79	31.20 ± 25.78	57.73 ± 31.65
Δ PI (%)	Mann–Whitney test, U = 539.000, Z = 0.834, r = 0.106, *p* = 0.404					
PPD (mm)	4.17 ± 0.94	2.49 ± 0.53	1.67 ± 0.96	4.43 ± 1.0	2.66 ± 0.54	1.76 ± 1.01
Δ PPD (mm)	Mann–Whitney test, U = 446.000, Z = −0.479, r = 0.061, *p* = 0.632					
BOP (%)	87.28 ± 18.41	22.97 ± 17.80	64.31 ± 24.17	87.47 ± 21.54	23.17 ± 15.52	64.30 ± 23.96
Δ BOP (%)	Mann–Whitney test, U = 472.000, Z = −0.113, r = 0.014, *p* = 0.910					
CAL (mm)	3.37 ± 1.72	2.09 ± 1.27	1.27 ± 0.78	3.17 ± 1.52	2.09 ± 1.02	1.07 ± 0.68
Δ CAL (mm)	Mann–Whitney test, U = 551.500, Z = 1.008, r = 0.128, *p* = 0.313					

SD—Standard deviation; PI—plaque index; PPD—periodontal probing depth; BOP—bleeding on probing; CAL—clinical attachment loss.

## Data Availability

The original contributions presented in this study are included in the article. Further inquiries can be directed to the corresponding author.

## Abbreviations

The following abbreviations are used in this manuscript:

BOP	Bleeding on probing
CAL	Clinical attachment loss
LAPP	Low-abrasive polishing powder
PI	Plaque index
PPD	Periodontal probing depth
SD	Standard deviation
SRP	Scaling and root planning
